# The association between acute gastrointestinal injury and mortality in elderly patients with gram-positive bacterial bloodstream infection in the intensive care unit: a retrospective 7-year study from a research hospital in China

**DOI:** 10.3389/fmed.2025.1634980

**Published:** 2025-09-23

**Authors:** Yuanqi Liang, Chulong Ma, Tingting Ma, Fan Lin, Tailin Guo

**Affiliations:** ^1^Department of Geriatric Medicine, Fujian Key Laboratory of Geriatrics Diseases, Fujian Provincial Center for Geriatrics, Provincial Clinical Medical College of Fujian Medical University, Fujian Provincial Hospital, Fuzhou University Affiliated Provincial Hospital, Fuzhou, China; ^2^Department of Respiratory and Critical Care Medicine, People's Hospital of Changji Hui Autonomous Prefecture, Changji, China; ^3^Department of General Practice, Provincial Clinical Medical College of Fujian Medical University, Fujian Provincial Hospital, Fuzhou University Affiliated Provincial Hospital, Fuzhou, China

**Keywords:** the elderly patients, gram-positive bacteria bloodstream infections, intensive care unit, mortality risk factors, acute gastrointestinal injury

## Abstract

**Background:**

Gram-positive bacterial bloodstream infections (GPB-BSI) are associated with high mortality in elderly ICU patients, yet prognostic factors remain understudied. Acute gastrointestinal injury (AGI), a common complication in critical illness, may exacerbate outcomes through gut-organ crosstalk. This study investigates the prognostic impact of AGI severity on 30-day mortality in elderly ICU patients with GPB-BSI.

**Methods:**

A single-center retrospective cohort study analyzed 117 ICU patients aged ≥60 years with culture-confirmed GPB-BSI (2018–2024). Data on demographics, microbiology, comorbidities, organ dysfunction, and antimicrobial therapy were collected. Multivariable Cox regression and ROC analyses assessed associations between AGI, clinical variables, and mortality.

**Results:**

The 30-day mortality rate was 50.4% (59/117). AGI severity independently predicted mortality: Grades I-II (aHR = 2.80, 95% CI = 1.05 ~ 7.46) and Grades III-IV (aHR = 6.89, 95%CI = 2.34 ~ 20.29). A combined SOFA-AGI score improved prognostic accuracy compared to SOFA score(AUC = 0.749 vs 0.729). Coagulase-negative staphylococci (60.3%) dominated isolates, predominantly hospital-acquired (79.4%) and catheter-related (47.0%). High resistance to penicillins (92.1%), fluoroquinolones (79.4%), and macrolides (77.0%) contrasted with retained susceptibility to linezolid (96.8%), tigecycline (92.9%), and vancomycin (94.4%).

**Conclusion:**

AGI severity is an independent predictor of mortality in elderly GPB-BSI patients. The diagnostic accuracy for mortality improves when gastrointestinal dysfunction assessment is incorporated into the SOFA score. These findings underscore the critical need for enhanced clinical attention to gastrointestinal function protection in geriatric critical ill patients.

## 1 Instruction

Bloodstream infections (BSIs), a prevalent clinical complication, pose substantial challenges in critical care management. These infections are especially common in geriatric intensive care unit (ICU) populations (1, 2), primarily attributed to immunosenescence and invasive interventions such as central venous catheterization, which constitutes significant risk factors for BSIs in ICU (1, 3). Epidemiological investigations consistently demonstrate that BSIs in ICU contribute to prolonged hospital stays, increased healthcare expenditures, and mortality rates ranging from 40 to 60% (3–5), particularly in advanced-age cohorts where immunocompromised status exacerbates clinical poor outcomes. Microbiological profiling reveals Gram-positive bacteria (GPB) as the predominant pathogens, with *Staphylococcus aureus* accounting for over 20% of culture-confirmed cases (6). Therefore, prognostic stratification and targeted management of GPB-associated bloodstream infections (GPB-BSI) in geriatric patients warrant prioritized clinical consideration.

Gastrointestinal dysfunction (AGI) represents a frequent comorbidity in critical care settings, with epidemiological studies indicating that >60% of ICU-admitted patients manifest clinically significant gastrointestinal disturbances (7, 8). Histologically confirmed intestinal epithelial damage has been documented in approximately 50% of ICU admissions (7). Multiple pathophysiological mechanisms contribute to AGI progression, including but not limited to: (1) sepsis-induced microcirculatory dysfunction, (2) hemodynamic instability in circulatory collapse, and (3) cytokine-mediated epithelial barrier disruption during systemic inflammation, collectively predisposing to intestinal ischemia–reperfusion injury and multiorgan failure. AGI severity demonstrates dose-dependent correlation with clinical outcomes, as evidenced by all-cause mortality rates reaching 28.1% in patients progressing to gastrointestinal failure (AGI grade III/IV) (9–11). These pathophysiological insights underscore the imperative for systematic AGI monitoring through validated scoring systems. The 2012 ESICM consensus guidelines operationalized AGI through a four-tier stratification system (Grades I-IV), where Grades I-II denote dysfunction and Grades III-IV constitute failure (10, 12).

Building upon the established mortality burden of GPB-BSI in ICU and the emerging evidence of pathophysiological interactions between AGI and systemic infections, this cohort study systematically investigates the prognostic impact of AGI severity (per ESICM criteria) on 30-day mortality in elderly (≥60 years) ICU patients with culture-confirmed GPB-BSI. The findings are expected to inform risk stratification frameworks and evidence-based interventions targeting gut-organ crosstalk in critical care.

## 2 Methods

### 2.1 Study design and patients

**Figure 1:**
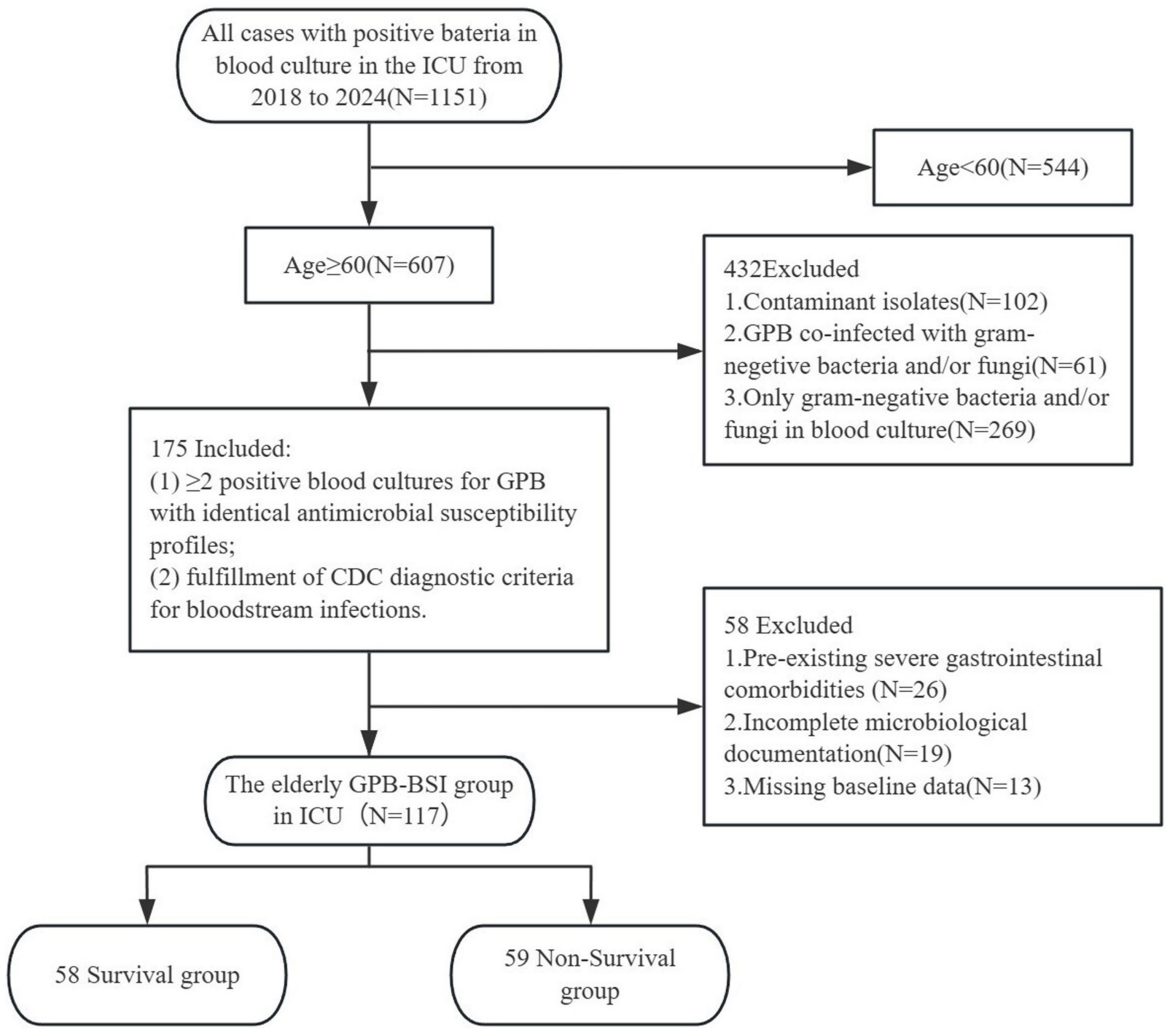
Flowchart.

This single-center retrospective cohort study was conducted in the ICU of Fujian Provincial Hospital, a tertiary academic medical center integrating clinical services, medical education, and translational research in Southeast China. We analyzed the data of patients aged 60 and older with blood cultures positive for GPB during their ICU stay from January 1, 2018, to December 31, 2024 (7-year observation period). Blood cultures followed standardized sepsis protocols requiring ≥2 sets collected aseptically from distinct anatomical sites within 24 h of suspected bacteremia. Inclusion required: (1) ≥ 2 positive blood cultures for GPB with identical antimicrobial susceptibility profiles, and (2) fulfillment of CDC diagnostic criteria for bloodstream infections. Exclusion criteria comprised: (1) age <60 years; (2) pre-existing severe gastrointestinal comorbidities (e.g., Crohn’s disease, GI malignancy); (3) incomplete microbiological documentation; (4) only gram-negative bacteria and/or fungi in blood culture; (5) co-infection with gram-negative bacteria and/or fungi; (6) contaminant isolates; (7) missing baseline data. In polymicrobial infections, the bacteremic episode was defined as the first culture-confirmed GPB-BSI occurrence preceding subsequent antimicrobial therapy. This study protocol was approved by the Institutional Review Board (No. k2024-06-031). Figure 1 illustrates the participant screening flowchart.

### 2.2 Data collection

Trained research doctors blinded to study hypotheses abstracted data from the hospital’s EHR system under IRB-approved protocols. Standardized case report forms captured: (1) Demographic parameters (age stratified as 60–74/≥75 years; biological sex); (2) Microbiological characteristics (pathogen identification per CLSI guidelines; contamination adjudication via CDC criteria); (3) Comorbidity burden; (4) Functional status (bedridden duration ≥14 days and long term hospitalization≥30 days before infection); (5) Infection epidemiology (community-onset vs. hospital-acquired); (6) Physiological severity (APACHE II and SOFA scores); (7) AGI staging; (8) Serial lactate measurements; (9) Ventilator-dependent days; (10) New-onset organ dysfunction; (11) Antimicrobial stewardship metrics (empirical vs. targeted therapy timelines). The primary endpoint was all-cause 30-day mortality from index bacteremia, ascertained through daily ICU rounds and structured telephone follow-up for discharged patients. For patients with bloodstream infection (BSI) present on ICU admission, APACHE II, SOFA scores were assessed serially within 72 h following ICU admission. For ICU-acquired BSI, APACHE II, SOFA score were assessed serially within 72 h following BSI diagnosis.

Among the covariates, indicators such as fluid balance, enteral nutrition strategies, and vasoactive drugs were not included. Because relevant detailed data were missing in electronic medical records, this precluded standardized quantification and inclusion in our regression models. To mitigate potential confounding, our models adjusted for acute kidney injury, SOFA/APACHE II, AGI grades (ESICM), which can serve as alternative indicators for fluid overload, the dosage of vasopressors, and the failure of enteral feeding.

### 2.3 Definition

BSI diagnosis adhered to the CDC surveillance definitions (13), which require: (1) Microbiological confirmation: For potential contaminants (e.g., coagulase-negative staphylococci), ≥2 concordant blood cultures drawn aseptically within 24 h per CLSI guidelines; (2) Clinical evidence: Either (a) core temperature >38.3 °C or <36.0 °C; (b) leukocyte count >12 × 10^9^/L or <4 × 10^9^/L; (c) systolic blood pressure <90 mmHg or mean arterial pressure (MAP) decrease >40 mmHg from baseline; (d) new-onset organ dysfunction attributable to infection.

Per the definition of Boev C et al. for infection classification (14), bacteremic episodes were categorized as: (1) Community-acquired: Positive blood culture obtained ≤48 h post-admission with pre-existing infection signs/symptoms documented in emergency department records; (2) Hospital-acquired: Blood culture positivity >48 h post-admission with: (a) No evidence of active infection on admission (C-reactive protein <10 mg/L, procalcitonin <0.5 ng/mL, and absence of fever/chills in triage notes); (b) Exclusion of incubating infections via structured symptom chronology review by two independent clinicians.

AGI is defined as newly acquired gut dysfunction secondary to acute systemic insults, characterized by impaired motility, barrier integrity, or absorptive capacity in critical illness. AGI was defined and graded strictly according to the 2012 criteria established by the Working Group on Abdominal Problems (WGAP) of the European Society of Intensive Care Medicine (ESICM) (Supplementary Table S1) (12). For patients with BSI present at ICU admission, AGI was calculated continuously within 72 h after admission to the ICU. For BSIs acquired during the ICU stay, AGI was assessed continuously within 72 h post-BSI diagnosis. AGI grades were determined based on objective parameters documented in the medical records. These included clinical symptoms (e.g., abdominal distension, vomiting, diarrhea, gastrointestinal bleeding), physical signs (e.g., abnormalities in bowel sounds), laboratory findings (e.g., positive fecal occult blood test), imaging findings (e.g., intestinal pneumatosis, air-fluid levels), nutrition support records (e.g., enteral nutrition held due to intolerance). All data extraction and AGI grading were performed independently by two trained critical care physicians. Cases with initial grading disagreements between the two physicians underwent review by a chief physician. A final consensus grade was reached through discussion to ensure maximum standardization in applying the assessment criteria. A random 20% subsample showed 92% inter-observer agreement.

Antimicrobial therapy was deemed inappropriate under three criteria: (1) delayed initiation (>6 h post blood culture collection); (2) discordance between empirical regimens and final antimicrobial susceptibility testing (AST) results; (3) persistent bacteremia despite ≥72 h of therapy.

Blood cultures were processed via the BacT/Alert 3D® system (bioMérieux, France) with standardized incubation protocols (aerobic/anaerobic bottles incubated at 35 °C for 5 days). Microbial identification and AST utilized the VITEK 2® platform (bioMérieux) with GNID/GPID cards, interpreted per CLSI 2023 breakpoints. Multidrug-resistant organisms (MDROs) were defined per 2012 ESCMID consensus (15): Gram-positive MDROs included methicillin-resistant *Staphylococcus aureus* (MRSA), vancomycin-resistant *Enterococcus* spp. (VRE), and methicillin-resistant coagulase-negative staphylococci (MRCNS) demonstrating non-susceptibility to ≥3 antimicrobial classes.

All protocols for bloodstream infection management and venous catheter care were implemented in accordance with China’s national guidelines and professional standards. Supplementary Tables S2, S3 shows the details (16–19). In cases of catheter-related infection involving *Staphylococcus aureus*, catheter removal is mandatory.

### 2.4 Statistical analysis

Statistical analyses were conducted with IBM SPSS Statistics 22.0 and R 4.3.1 (R Foundation). Normally distributed continuous variables are reported as mean ± standard deviation (SD) and analyzed with independent samples t-test. Non-parametric variables are summarized as median [interquartile range, IQR] and compared via Mann–Whitney U test. Categorical variables are expressed as counts (percentages) and evaluated by Pearson’s χ^2^ test or Fisher’s exact test (for expected cell counts <5). Statistical significance was defined as two-tailed *p* < 0.05.

Multivariable Cox proportional hazards regression with backward elimination was implemented to quantify the independent association between AGI severity and 30-day mortality in GPB-BSI, expressed as adjusted hazard ratios (HR) with 95% confidence intervals (95%CI). Four hierarchical models were developed: Model 1 included only AGI; Model 2 adjusted for age, sex, comorbidities, MDROs; Model 3 further adjusted for irrational antibiotic use, antibiotic combination (>3 types), mechanical ventilation, infection timing, long-term bedridden patients, long term hospitalization; and Model 4 further adjusted for organ failure. Covariates were selected based on prior findings, clinical significance, or a *p*-value <0.05 in univariate analysis. Fine-Gray subdistribution hazard models were used to verify the robustness of the results to address competing mortality risks. The association between AGI and 30-day mortality was verified across subgroups to check for correlations within each. Receiver operator characteristic curves (ROC) were used to evaluate the predictive value of Acute Physiology and Chronic Health Evaluation (APACHE II), Sequential Organ Failure Assessment (SOFA), AGI scores, lactic acid (Lac), and SOFA-AGI combined scores for adverse outcomes, assessing the area under the curve (AUC), optimal cutoff values, specificity, and sensitivity. Groups were stratified using the optimal cutoff for SOFA-AGI scores. Kaplan–Meier estimators assessed outcome incidence, and log-rank tests compared groups. A two-tailed *p*-value <0.05 was considered statistically significant.

## 3 Results

### 3.1 The composition of GPB isolated from BSI patients and the sources of infection

**Figure 2:**
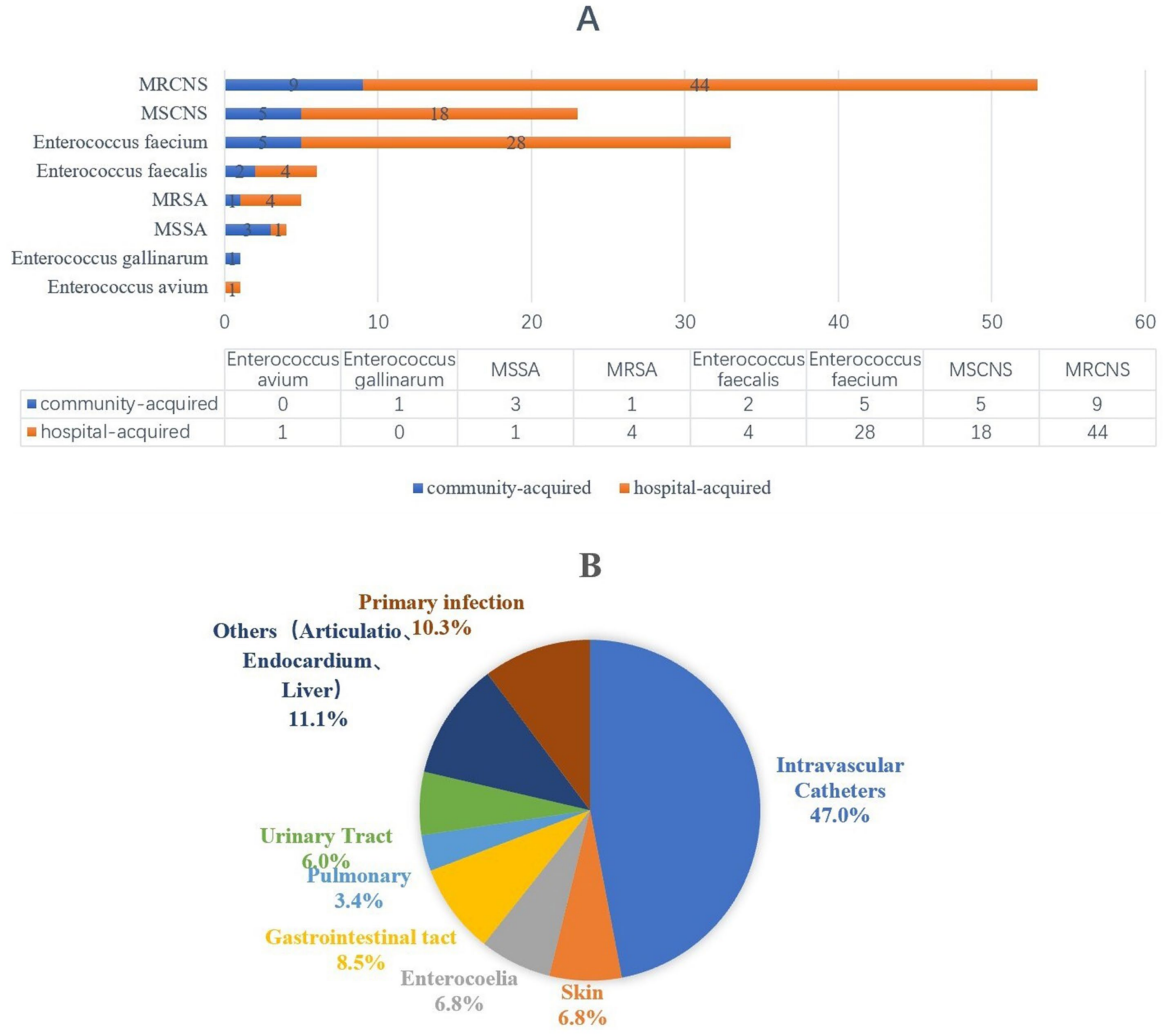
The distribution of pathongen and infection sources among patients with bloodstream infection. **(A)** Pathongen distribution. **(B)** The sources of infection.

Among 117 evaluable patients, 112 (95.7%) presented with monomicrobial bacteremia, while 5 (4.7%) demonstrated two types of GPB. Microbiological analysis identified 126 gram-positive isolates, with 100 (79.4%) demonstrating hospital-acquired infection patterns. The predominant etiological agents were coagulase-negative *Staphylococcus* spp. (CoNS, 76, 60.3%), *Enterococcus* spp. (39, 31.0%), and *Staphylococcus aureus* (9, 7.1%). As detailed in Figure 2A, the pathogen distribution revealed MRCNS as the dominant GPB-BSI agents. Figure 2B demonstrates that indwelling vascular catheters constituted the primary infection focus (55/117, 47.0%).

### 3.2 Antimicrobial resistance analysis of pathogens

Table 1 delineates the antimicrobial resistance patterns of predominant pathogens. Among 126 isolates, 58 (46.0%) exhibited methicillin resistance: 5 MRSA (4.0%) and 53 MRCNS (42.1%). A single VRE isolate (0.8%) was identified from fecal enterococcus strain. GPB isolates retained high susceptibility to linezolid (96.8%), tigecycline (92.9%), and vancomycin (94.4%). Conversely, resistance rates surpassed 50% for penicillin antibiotics (92.1%), fluoroquinolones (79.4%), aminoglycoside (54.8%) and macrolides (77.0%). Nosocomial strains showed susceptibility (>50%) to linezolid (96.0%), tigecycline (91.0%), vancomycin (94.0%), tetracyclines (61.0%). Community-onset strains showed susceptibility (>50%) to linezolid (96.2%), tigecycline (100.0%), vancomycin (96.2%), aminoglycoside (73.1%), quinolone (73.1%), tetracyclines (53.8%), and aminoglycosides (53.8%). Extended susceptibility data stratified by pathogen species and infection source are available in Supplementary Tables S4–S6.

**Table 1:** Analysis of drug resistance in common pathogenic bacteria.

Antibiotics	MRSA (*n* = 5)	MSSA (*n* = 4)	MRCNS (*n* = 53)	MSCNS (*n* = 23)	*Enterococcus faecium* (*n* = 33)	*Enterococcus faecalis* (*n* = 6)
Penicillin G	5 (100.0)	4 (100.0)	52 (98.1)	21 (91.3)	31 (93.9)	1 (16.7)
Oxacillin	5 (100.0)	0	50 (94.3)	16 (69.6)	30 (90.9)	3 (50.0)
Ampicillin	3 (60.0)	0	38 (71.7)	11 (47.8)	30 (90.9)	1 (16.7)
Chloramphenicol	3 (60.0)	0	37 (69.8)	12 (52.2)	29 (87.9)	4 (66.7)
Ciprofloxacin	5 (100.0)	1 (25)	43 (81.1)	16 (69.6)	32 (97.0)	3 (50.0)
Levofloxacin	5 (100.0)	1 (25)	43 (81.1)	14 (60.9)	31 (93.9)	2 (33.3)
Moxifloxacin	4 (80.0)	4 (100)	35 (66.0)	7 (30.4)	31 (93.9)	2 (33.3)
Rifampicin	5 (100.0)	0	12 (22.6)	4 (17.4)	27 (81.8)	3 (50.0)
SMZ	1 (20.0)	0	22 (41.5)	8 (34.8)	27 (81.8)	3 (50.0)
Clindamycin	3 (60.0)	0	35 (66.0)	10 (43.5)	29 (87.9)	3 (50.0)
Erythrocin	4 (80.0)	0	42 (79.2)	13 (56.5)	32 (97.0)	4 (66.7)
Streptomycin	3 (60.0)	0	36 (67.9)	10 (43.5)	19 (57.6)	4 (66.7)
Vancomycin	5 (100.0)	0	1 (1.9)	0	1 (3.0)	0
Quinupristin/ Dalfopristin	5 (100.0)	0	6 (11.3)	1 (4.3)	2 (6.1)	6 (100.0)
Gentamicin	4 (80.0)	0	28 (52.8)	12 (52.2)	21 (63.6)	3 (50.0)
Linezolid	0	0	4 (7.5)	0	1 (3.0)	0
Tetracycline	3 (60.0)	2 (50)	19 (35.8)	2 (8.7)	19 (57.6)	5 (83.3)
Tigecycline	1 (20.0)	0	5 (9.4)	1 (4.3)	2 (6.1)	0.000

### 3.3 Analysis of clinical characteristics of elderly GPB-BSI patients in the ICU

This study enrolled 117 geriatric ICU patients (median age 75 years, IQR 66–84) with culture-confirmed GPB-BSI, all meeting BSI diagnostic criteria. The baseline analysis is detailed in Table 2. Sepsis-associated organ dysfunction developed in 110 patients (94.0%), day all-cause ICU mortality occurring in 59 cases (50.4%), BSI-associated mortality occurring in 42 (35.9%). Table 2 delineates comparative analyses between death group (*n* = 59) and survival group (*n* = 58), stratified by baseline characteristics and therapeutic interventions. Demographic parameters (age, sex distribution) and comorbidity burden showed no intergroup disparities (all *p* > 0.20). Irrational antibiotic therapy, mechanical ventilation (<72 h), acute renal failure, septic shock, and AGI were different between the two groups (all *p* < 0.10). Multidrug-resistant pathogen, infection timing, antibiotic combination (>3 types), long term hospitalization, long-term bedridden patients, acute heart failure, and acute respiratory failure did not demonstrate prognostic significance.

**Table 2:** Univariate analysis of mortality in elderly ICU patients with gram-positive bacterial bloodstream infections.

Characteristics	Total (*n* = 117)	Survival group (*n* = 58)	Death group (*n* = 59)	*p*
Gender				
Male	85 (72.6)	41 (70.7)	44 (74.6)	0.637
Female	32 (27.4)	17 (29.3)	15 (25.4)	
Age	75 (66, 84)	75 (60, 85)	75 (66, 84)	0.963
Age<75	58 (49.6)	30 (51.7)	28 (47.5)	0.644
Age≥75	59 (50.4)	28 (48.3)	31 (52.5)	
MDROs	57 (48.7)	28 (48.3)	29 (49.2)	0.924
Infection timing				
Hospital-acquired infection	94 (80.3)	47 (81)	47 (79.7)	0.852
Community-acquired infection	23 (19.7)	11 (19)	12 (20.3)	
Comorbidities				
Comorbidities≥3	98 (83.8)	50 (86.2)	48 (81.4)	0.477
Digestive diseases	51 (43.6)	27 (46.6)	24 (40.7)	0.522
Cancer	37 (31.6)	17 (29.3)	20 (33.9)	0.594
Type 2 Diabetes	30 (25.6)	18 (31)	12 (20.3)	0.185
Antibiotic combination (>3 types)	67 (57.3)	34 (58.6)	33 (55.9)	0.769
Irrational antibiotic therapy	36 (30.8)	13 (22.4)	23 (39)	0.052
Long term hospitalization	64 (54.7)	30 (51.7)	34 (57.6)	0.521
Long-term bedridden patients	60 (51.3)	27 (46.6)	33 (55.9)	0.310
Mechanical ventilation (<72 h)	49 (41.9)	31 (53.4)	18 (30.5)	0.012
Organ dysfunction post-BSI				
MODS> = 3	64 (54.7)	23 (39.7)	41 (69.5)	0.001
Acute heart failure	71 (60.7)	32 (55.2)	39 (66.1)	0.226
Acute respiratory failure	52 (44.4)	27 (46.6)	25 (42.4)	0.649
Acute renal failure	60 (51.3)	23 (39.7)	37 (62.7)	0.013
Septic shock	51 (43.6)	17 (29.3)	34 (57.6)	0.002
AGI	14 (12.0)	2 (3.4)	12 (20.3)	0.005
AGI I-II	7 (6.0)	1 (1.7)	6 (10.2)	0.028
AGI III-IV	7 (6.0)	1 (1.7)	6 (10.2)	

**Table 3:** Relationship between GPB-BSI combined with AGI and poor prognosis within 30 days in elderly ICU patients.

Groups	*N* (%)	Model1	Model2	Model3	Model4
		HR (95% CI)	HR (95%CI)	HR (95%CI)	HR (95%CI)
AGI	12 (85.7)	3.27 (1.72 ~ 6.21) *	4.15 (2.04 ~ 8.43) *	3.58 (1.72 ~ 7.47) *	2.74 (1.27 ~ 5.94) ***
Subgroups					
AGI 0	47 (45.6)	1 (Ref)	1 (Ref)	1 (Ref)	1 (Ref)
AGI I-II	6 (85.7)	2.63 (1.12 ~ 6.17) ***	3.31 (1.3 ~ 8.48) ***	3.31 (1.26 ~ 8.69) ***	2.80 (1.05 ~ 7.46) ***
AGI III-IV	6 (85.7)	4.37 (1.83 ~ 10.43) *	4.93 (1.91 ~ 12.72) *	5.57 (1.97 ~ 15.73) *	6.89 (2.34 ~ 20.29) *
*p*-for trend		<0.001	<0.001	<0.001	<0.001

*p ≤ 0.001,**p < 0.010,***p < 0.05; Model 1: No adjustment; Model 2: age, gender, Comorbidities (digestive diseases, cancer, type 2 Diabetes), MDROs; Model 3: age, gender, comorbidities (digestive diseases, cancer, type 2 diabetes), MDROs, antibiotic combination (>3 types), irrational antibiotic therapy, mechanical ventilation (<72 h), infection timing, long-term bedridden patients, long term hospitalization; Model 4: age, gender, comorbidities (digestive diseases, cancer, type 2 diabetes), MDROs, antibiotic combination (>3 types), irrational antibiotic therapy, mechanical ventilation (<72 h), infection timing, long-term bedridden patients, long term hospitalization, acute respiratory failure, acute heart failure, acute renal failure, septic shock.

### 3.4 Relationship between AGI and 30-day-death

Multivariable Cox regression adjusted for age, organ failure, and antimicrobial stewardship adherence identified AGI severity as an independent mortality predictor [aHR (95%CI) 2.74 (1.27 ~ 5.94), *p* = 0.011] (Table 3). AGI grade I-II demonstrated an adjusted hazard ratio (aHR) of 2.80 (95%CI 1.05 ~ 7.46, *p* = 0.040), while AGI III-IV showed an aHR of 6.89 (95%CI 2.34 ~ 20.29, *p* < 0.001) relative to AGI-free controls, with significant gradient between grades (*p* < 0.001). Notably, this dose–response pattern persisted across incremental adjustment models (Model 1–4: aHR range 2.63–6.89, all *p* < 0.05).

### 3.5 Sensitivity analysis and subgroup analysis

To address competing mortality risks (17/117, 14.5% non-BSIrelated deaths), we employed Fine-Gray subdistribution hazard model, which confirmed temporal stability of the AGI-mortality association (sHR3.29, 95%CI 1.65 ~ 6.56, *p* < 0.001). Analyses using model4 yielded concordant results (HR 2.74 vs. sHR 3.94, Gray’s test *p* = 0.001) (Supplementary Table S7).

**Figure 3:**
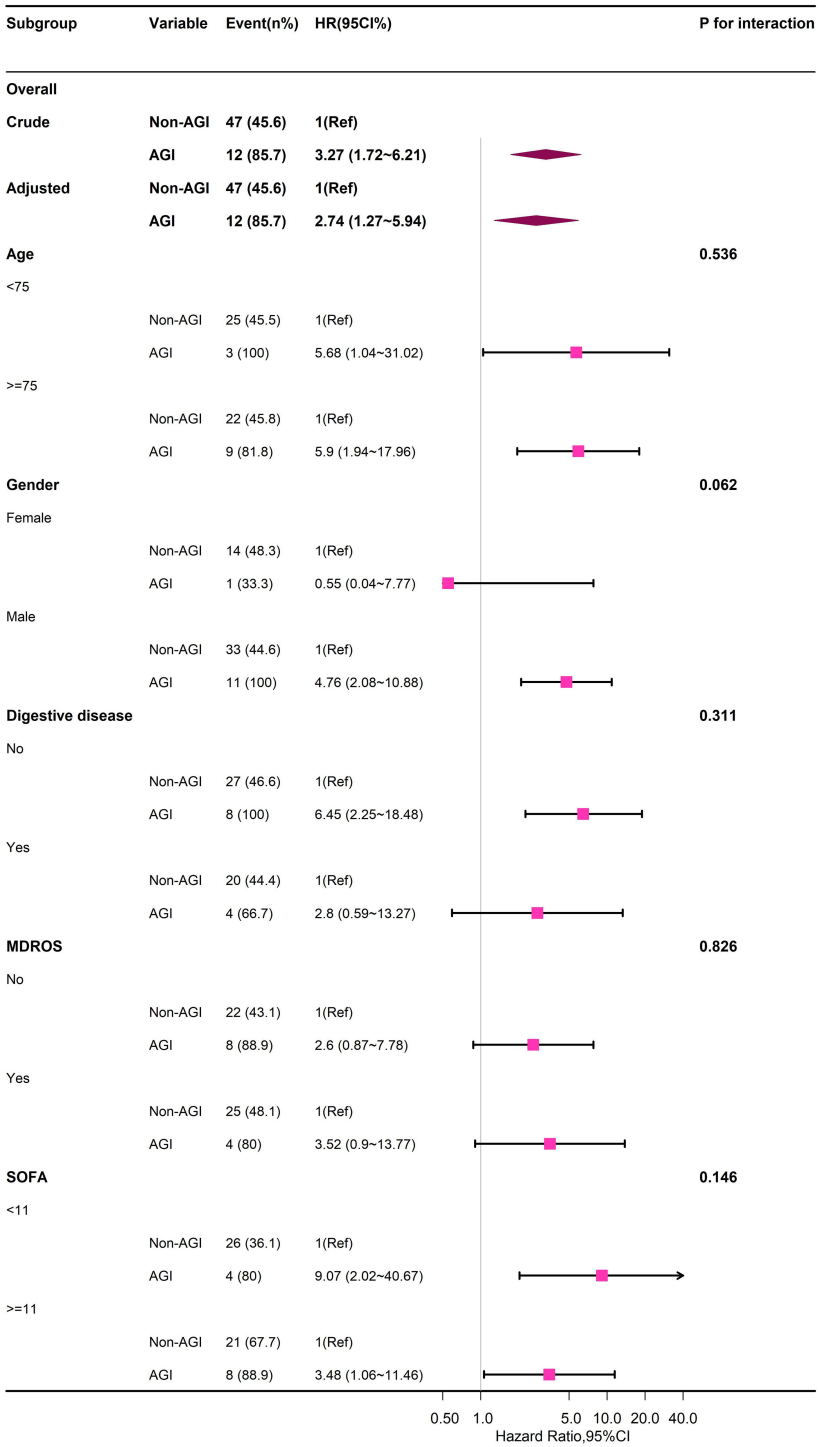
Forest plot of multivariable COX analysis between AGI and 30-day-death in older patients with GPB-BSI.

Stratified analyses across multiple subgroups revealed no significant interactions in any subgroup when stratified by gender, age, digestive diseases, MDROS, and SOFA (Figure 3; Supplementary Table S8).

### 3.6 APACHE II, SOF, Lac, and AGI score for predicting adverse prognosis of bloodstream infections

**Figure 4:**
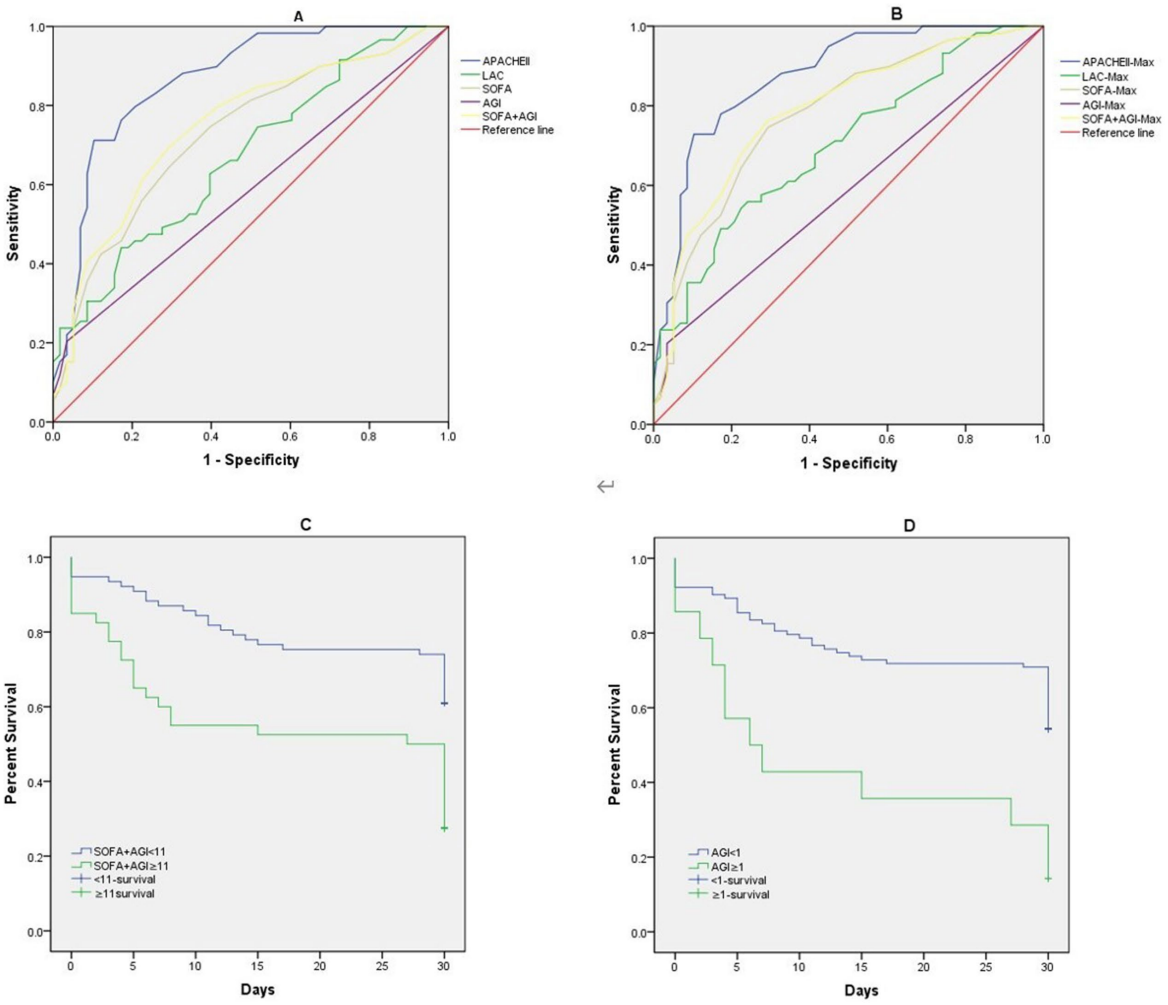
Receiver operating characteristic curves of APACHE II, SOFA, AGI, SOFA+AGI, Lac, **(A)** APACHE II, SOFA, AGI, SOFA+AGI, Lac on the first day of BSI diagnosis; **(B)** Maximum APACHE II, SOFA, AGI, SOFA+AGI, Lac within 72 h of BSI diagnosis; Kaplan–Meier curves estimate of probabilities of survival among patients who had different AGI score and SOFA-AGI score. **(C)** Probabilities of survival among patients who SOFA-AGI ≥ 11 or not((Log-rank *p* < 0.001)). **(D)** Probabilities of survival among patients who AGI ≥ 1 or not((Log-rank *p* < 0.001)).

Table 4 illustrates the predictive value of the APACHE II score, SOFA score, AGI grade, SOFA + AGI score, and Lac levels within the first 24 h for mortality in elderly patients with GPB-BSI. The AUC values for AGI grade and SOFA + AGI score were 0.585 and 0.749, respectively. The ROC curves for the highest values of these indicators within 72 h of BSI diagnosis were similar to those within the first 24 h (Figures 4A,B).

**Table 4:** Diagnostic efficacy of APACHE II, SOFA, AGI, SOFA+AGI scores, and lactate for poor prognosis in GPB-BSI.

Scoring system	AUC	*p*	95% CI	Best cut-off	Sensitivity	Specificity
APACHEII	0.867	0.000	0.801–0.933	26.500	0.763	0.828
SOFA	0.729	0.000	0.638–0.820	11.500	0.424	0.879
LAC	0.672	0.001	0.576–0.768	3.550	0.441	0.828
AGI score	0.585	0.111	0.482–0.689	0.500	0.203	0.966
SOFA+AGI	0.749	0.000	0.660–0.838	10.500	0.492	0.828

Figures 4C,D demonstrate that Kaplan–Meier analysis revealed significantly lower survival rates in patients with AGI scores ≥1 and SOFA + AGI scores ≥11 (Log-rank *p* < 0.001).

## 4 Discussion

BSIs continue to pose substantial mortality risks in critical care settings, particularly among vulnerable geriatric populations. This retrospective cohort study of 117 elderly ICU patients with GPB-BSI revealed a 30-day all-cause mortality rate of 50.4% (59/117). Coagulase-negative *Staphylococcus* spp. predominated (60.3%), followed by *Enterococcus* spp. (31.0%) an *S. aureus* (7.1%). Sepsis-induced multiorgan dysfunction affected 94% of patients, while 12.0% of them developed acute gastrointestinal injury (AGI). Multivariable Cox regression adjusted for covariates such as age, organ failure, and antimicrobial stewardship identified AGI severity as an independent mortality predictor. Stratification by SOFA-AGI composite scores demonstrated superior prognostic accuracy. Sensitivity analyses using competing risk regression and subgroup analysis confirmed model robustness.

In this study, the most common pathogen was coagulase-negative *Staphylococcus* spp. (60.3%), with 47.0% of patients linked to catheter-related infections. Multiple retrospective cohort studies in US have found that over 70% of ICU bloodstream infections were secondary and catheter-associated, with *Staphylococcus aureus* accounting for 40% of GPB (6, 20). In some Asian countries, *Staphylococcus epidermidis* constitutes 40 to 60% of ICU BSI pathogens (21, 22), and central venous catheters are key sources of coagulase-negative staphylococci (23), which were consistent with our findings. The antibiotic resistance of ICU BSI isolates has been increasingly reported (24). Perez-Crespo et al. (25) noted that hospital-acquired BSI in Spanish ICUs frequently were caused by multidrug-resistant pathogens, similar to reports from Qatar hospitals (26). In our study, 48.7% of patients were infected with MDROs. Our antimicrobial susceptibility results indicate that GPB exhibited high susceptibility exclusively to linezolid, tigecycline, and vancomycin, while resistance to commonly used antibiotics such as penicillins, quinolones, and macrolides surpassed 50%. Ejaz et al. (21) also reported 100% susceptibility of GPB isolates from ICU infections to these three drugs. Cairns, et al. (27) observed substantial geographic variation in antibiotic resistance rates. For instance, vancomycin resistance rates exceeded 40% in some developed countries, while vancomycin-resistant enterococci (VRE) prevalence in Asia ranged from 5 to 20%. This variation likely stems from multiple factors, including differential access to effective antibiotics, variations in antimicrobial stewardship programs, prescribing practices among clinicians, patient adherence behaviors, incomplete antibiotic supply chains, and divergent medication usage patterns across countries (28). These factors can significantly impact susceptibility testing results. While the susceptibility results in our study have regional restrictions, it broadly aligns with the current antibiotic resistance profile of GPB in China (29, 30).

This study focused on elderly ICU patients (median age 75 years), in whom hospital-acquired bloodstream infections (HA-BSI) accounted for 80.3% of cases. As illustrated in Figure 2B, nearly half (47%) of GPB-BSI were associated with the use of intravascular catheters. Therefore, the HA-BSI prevention lies in the effective prevention of catheter-related bloodstream infections (CRBSI). As the meta-analysis by Huang et al., risk factors for central CRBSI can be categorized into modifiable and non-modifiable factors (31). In our cohort, advanced age, extended antibiotic use and multimorbidit constituted non-modifiable risks. The decline in physiological reserve and presence of immunosenescence in elderly patients predispose them to increased risks of multidrug-resistant organism infections and invasive infections (32). Prolonged hospitalization (54.7%) as a modifiable risk factor also significantly associated with an increased risk of HA-BSI. Huang et al. (31) indicated that other modifiable factors—including extended catheter indwelling duration, use of multi-lumen catheters, administration of total parenteral nutrition, and repeated catheterization—may elevate the risk of HA-BSI by exacerbating systemic inflammation, intestinal dysfunction, and gut microbiota translocation, also potentially contributing to the development of AGI. However, this retrospective study lacks detailed metrics on catheter management and parenteral nutrition practices, representing a limitation. Future prospective studies should incorporate these specific indicators to better elucidate their interplay between AGI and mortality in elderly patients with GPB-BSI. Although we strictly adhered to Chinese guidelines for catheter management, certain discrepancies with the latest WHO global guidelines (2024), (33)—such as preferences in skin antisepsis, catheter selection, dressing management and lock therapy—may represent another modifiable aspect affecting infection outcomes. Moving forward, stringent infection prevention strategies—including prompt removal of unnecessary catheters, strict adherence to aseptic techniques, and updating institutional protocols to align with international standards—combined with antimicrobial reasonable use in the context of interdisciplinary collaboration, is essential to reduce the incidence of HA-BSI and improve patient outcomes.

While MODS and septic shock have well-documented associations with increased mortality, only AGI showed a consistent independent association with mortality in this study. Previous studies have shown that AGI increases the risk of death (aOR = 1.224) (10). However, this study is the first to establish this association in elderly patients with GPB bloodstream infections, and this increased risk is likely attributable to the advanced age of the patient cohort. This finding highlighted that AGI may be a key independent risk factor for poor prognosis in elderly ICU patients with GPB bloodstream infections.

Our study identified only 14 AGI cases (12.0% of the cohort), including 7 Grade I-II and 7 Grade III-IV events. This limited sample size, particularly for higher-grade AGI (III-IV), inherently constrains the precision of our graded risk estimates. Despite limited cases, AGI severity showed a graded mortality risk (I-II: aHR = 2.80; III-IV: aHR = 6.89; *p* for trend < 0.001), aligning with established pathophysiology where gut failure exacerbates sepsis outcomes (9, 10). The AGI-mortality association persisted across four adjustment models (Model 1–4), with hazard ratios remaining significant even after accounting for organ failures and antimicrobial stewardship. Our results concord with prior studies in broader ICU populations. For example, Hu et al. (34) reported a 2.8-fold mortality increase per AGI grade, while Reintam et al. (11) documented 40% mortality in gastrointestinal dysfunction group (*n* = 60 cases). To mitigate small-sample bias, we employed competing risk regression confirmed AGI’s temporal association with mortality (sHR = 3.94, 95%CI = 1.96–7.92; *p* < 0.001), reducing confounding from non-BSI deaths. Otherwise, stratified sensitivity analyses showed no subgroup heterogeneity (*p*-interaction>0.05 for all subgroups in Figure 3), suggesting generalizability within constraints. While our graded AGI analysis should be interpreted cautiously, it provides preliminary evidence that even mild AGI (Grade I-II) significantly impacts GPB-BSI outcomes—a novel insight warranting validation. Future multicenter studies with larger AGI cohorts are needed to verify this finding.

In this study of elderly patients with GPB-BSI, the majority (>90%) developed at least one organ dysfunction and over 40% experienced septic shock. The gut, a highly vulnerable organ, is among the earliest organs affected in sepsis and acts as a critical indicator of disease severity in critically ill patients (10, 11). Research by Bruno Monteiro Pereira et al. has shown a direct association between sepsis and AGI, where the risk of AGI increases significantly when SOFA scores ≥ 7 (35). This increase in AGI risk is attributed to stress-mediated inflammatory responses, leading to increased vascular permeability, extravascular fluid accumulation, intestinal interstitial edema, and local hypoperfusion (10). These pathophysiological changes can manifest as AGI symptoms, including diarrhea, hematochezia, abdominal distension, dyspepsia, feeding intolerance, intra-abdominal hypertension, and even abdominal compartment syndrome.

Current treatment strategies for septic shock, beyond antimicrobial therapy, are primarily centered on fluid resuscitation and vasopressor administration to manage distributive hypoperfusion. However, these interventions are associated with an increased risk of developing intra-abdominal hypertension (IAH) and abdominal compartment syndrome (ACS) (10, 35). Additionally, early initiation of immunomodulatory short-peptide enteral nutrition can help mitigate the severity of AGI-related complications (36). However, in the regression analysis of this study, no adjustments were made to indicators such as fluid balance, enteral nutrition strategies, and vasoactive drugs. Particularly cumulative fluid balance and enteral nutrition adequacy—may theoretically confound the AGI-mortality association via two pathways: Fluid overload exacerbates bowel wall edema, potentially misclassifying AGI severity (37). Enteral nutrition failure (e.g., <20% calorie target) may prolong AGI duration (8), creating residual confounding where malnutrition drives mortality independently. Notably, our stratified analyses demonstrated consistent AGI-mortality associations across subgroups, suggesting robustness despite unmeasured variables. Furthermore, AGI severity grading inherently reflects downstream effects of fluid/nutrition management (e.g., enteral intolerance in AGI II-IV). We acknowledge that positive fluid balance and delayed enteral nutrition may exacerbate AGI. However, such factors likely act as mediators (not confounders) on the causal pathway between critical illness and AGI. Adjusting for mediators would bias the estimated direct effect of AGI. In the future we can conduct AGI prospective studies including standardize data collection on fluid management, vasopressor titration, and nutrition support.

The severity of AGI exhibits a strong correlation with mortality, and ACS is associated with a 40% 30-day mortality rate (10). While AGI is unlikely to be a direct cause of death, it significantly influences patient outcomes. As the largest immune organ, the gut plays a critical role in the prognosis of BSI through its barrier function. AGI-induced intestinal mucosal ischemia, epithelial cell apoptosis, and disruption of tight junctions can promote translocation of gut bacteria and endotoxins into the systemic circulation. This process not only exacerbates systemic inflammatory response syndrome (SIRS) but may also increase the pathogen burden of the primary BSI through mechanisms like pathogen molecular mimicry and immune evasion. In elderly ICU patients, age-associated gut dysbiosis and immunosenescence further amplify this risk, creating a vicious cycle (38). Animal studies directly demonstrate the detrimental impact of gut microbiota on outcomes following intestinal injury. Wang et al. (39) found that gut microbiota and their metabolic byproduct, succinate, promoted alveolar macrophage polarization, alveolar epithelial apoptosis, and lung injury during intestinal ischemia/reperfusion, thereby accelerating mortality. Another study showed that pre-treatment with probiotics prevented injury-induced bacterial translocation, reduced pro-inflammatory cytokine release and endotoxin levels, decreased intestinal epithelial cell apoptosis, and ultimately lowered mortality in mice (40). Consequently, future studies should integrate longitudinal monitoring of gut function and microbiota dynamics in animal models to quantify the impact of the gut-organ axis on clinical outcomes.

The International AGI Grading System (IAGIS), widely adopted for its simplicity in assessing gastrointestinal injury severity, has demonstrated clinical utility in critically ill populations. Zhang et al. (41) validated its prognostic utility in evaluating critically ill patients with gastrointestinal dysfunction. However, since AGI grading only partially and indirectly reflects the status of a single organ system, both SOFA and APACHE II scores demonstrated superior predictive accuracy for mortality in elderly patients with severe GPB-BSI compared to AGI alone. Existing studies indicate that APACHE IV offers better predictive value for mortality than APACHE II (42, 43). However, our study did not collect APACHE IV data for a direct statistical comparison. Given that the SOFA score focuses on dynamic monitoring of multiple organ systems, providing a comprehensive assessment of a patient’s clinical course and status changes, we hypothesized that combining the AGI score with SOFA might optimize predictive capability. This study confirmed that a combined SOFA-AGI score demonstrates unique clinical relevance in elderly ICU patients with GPB-BSI. We found that the SOFA-AGI score (AUC = 0.749) demonstrated superior discriminatory ability compared to the SOFA score alone (AUC = 0.729), suggesting that AGI provides incremental predictive value in this subpopulation second only to the APACHE II score (AUC = 0.867), despite requiring fewer variables. This finding aligns with prior studies validating gastrointestinal-integrated scores (8, 9). Unlike APACHE II/IV scores, SOFA-AGI specifically integrates AGI, a previously underappreciated prognostic factor in bloodstream infection. This targeted focus on gut-organ crosstalk is consistent with emerging evidence that gastrointestinal injury can exacerbate sepsis outcomes via bacterial translocation and immune dysregulation (38). Unique Advantages of SOFA-AGI over APACHE II/IV: First, AGI grading directly reflects intestinal function integrity, a key determinant of secondary infections in bacteremia (10, 11). Second, SOFA is routinely collected globally in ICUs. Adding AGI assessment requires minimal additional training (12), whereas APACHE II/IV involves complex variables less feasible in resource-limited settings. Third, identifying AGI suggests actionable interventions (e.g., enteral nutrition optimization, fluid balance management (36)), whereas APACHE II/IV scores are primarily used for prognostication. For clinicians managing elderly GPB-BSI patients, SOFA-AGI offers a practical risk stratification tool. At a cutoff value≥11, it identifies high-risk patients with significantly reduced survival (Log-rank *p* < 0.001), enabling the early initiation of gut-protective strategies (e.g., immunomodulatory enteral nutrition (36)). In contrast, the APACHE II/IV scoring systems lack specific guidance for organ-targeted supportive interventions. Therefore, we believe that integrating AGI into sepsis management protocols may more effectively improve outcomes in this vulnerable population than complex scoring systems alone. Future multicenter studies should prioritize comparing SOFA-AGI with APACHE II/IV to validate its utility across diverse settings.

This study is the first to establish a significant association between gastrointestinal dysfunction and prognosis in elderly patients with severe GPB-BSI. The diagnostic accuracy for mortality improves when gastrointestinal dysfunction assessment is incorporated into the SOFA score. These findings underscore the critical need for enhanced clinical attention to gastrointestinal function protection in geriatric critical ill patients.

However, the study has several limitations. First, as a retrospective study conducted in a single-center ICU in southeastern China, the enrolled elderly patients (≥60 years) were predominantly from tertiary hospitals in Fujian Province. Consequently, their baseline characteristics—including comorbidity profiles and carriage rates of resistant bacteria—may differ from those in other Chinese regions. Heterogeneity in infection control resources and antimicrobial stewardship intensity likely exists across healthcare settings. The generalizability of our resistance data requires further validation in diverse environments. Second, the assessment of AGI was dependent on the completeness and accuracy of the medical records. Documentation of symptoms and signs could vary in detail among different clinicians, with a focus on feeding intolerance—a symptom that is vaguely defined and influenced by local feeding protocols and its management. Potentially introducing information bias, despite the use of designated assessors. Nonetheless, multiple studies have demonstrated the clinical utility of AGI grading in mortality prediction (43, 44). Future prospective studies should implement a rigorous AGI assessment protocol, including standardized rater training and inter-rater reliability testing. Third, we did not conduct a formal inter-rater reliability test to quantify the degree of agreement among the assessors. However, by randomly selecting 20% of the assessment results, we found that only 8% were inconsistent, and these were then calibrated by the chief physician. Forth, the sample size is relatively small, especially with respect to patients with advanced gastrointestinal failure, potentially overestimating effect sizes due to small event numbers. However, the results remained robust across adjustments for different covariates and sensitivity analyses, suggesting generalizability within the constraints of the study. Fifth, the lack of data on fluid balance status, treatment protocols, catheter care and nutritional support strategies introduces residual confounding risks. Despite adjusting for known confounders across multiple models and achieving consistent results, this limitation persists. Sixth, while this study identified an association between AGI and mortality risk in GPB-BSI, it did not explore the underlying mechanisms. Future research should integrate longitudinal monitoring of gut function dynamics in animal models to quantify the clinical impact of the gut-organ axis. Future studies validating the association between AGI scores and patient prognosis should involve large-scale, prospective, multicenter trials in different regions and different age groups to confirm its clinical utility and reproducibility.

## Data Availability

The raw data supporting the conclusions of this article will be made available by the authors, without undue reservation.
