# Supplementary material for: The association between acute gastrointestinal injury and mortality in elderly patients with gram-positive bacterial bloodstream infection in the intensive care unit: a retrospective 7-year study from a research hospital in China
Source: Front Med (Lausanne). 2025 Sep 23;12:1634980. doi: 10.3389/fmed.2025.1634980 (PMC12500591; doi:10.3389/fmed.2025.1634980)
Supplement: Supplementary file 1 [file Data_Sheet_1.docx]

**Supplemental Table S1**. Classification of AGI

| Grade | Definition |
| --- | --- |
| I | The function of the gastrointestinal tract is partially impaired, expressed as gastrointestinal symptoms related to a known cause, and perceived as transient. Examples: postoperative nausea and/or vomiting during the first days after abdominal surgery, postoperative absence of bowel sounds, diminished bowel motility in the early phase of shock. |
| II | The gastrointestinal tract is not able to perform digestion and absorption adequately to satisfy the nutrient and fluid requirements of the body. There are no changes in general condition of the patient related to gastrointestinal problems. Examples: gastroparesis with high gastric residuals or reflux, paralysis of the lower GI tract, diarrhea, intra-abdominal hypertension (IAH) grade I (intra-abdominal pressure [IAP] 12-15 mm Hg), visible blood in gastric content or stool. Feeding intolerance is present if at least 20 kcal/kg BW per day via enteral route cannot be reached within 72 h of feeding attempt. |
| III | Loss of gastrointestinal function, and restoration of gastrointestinal function is not achieved despite interventions, and the general condition is not improving. Examples: Despite treatment, feeding intolerance is persisting—high gastric residuals, persisting GI paralysis, occurrence or worsening of bowel dilatation, progression of IAH to grade II (IAP 15-20 mm Hg), low abdominal perfusion pressure (APP) (below 60 mmHg). Feeding intolerance is present and possibly associated with persistence or worsening of multiple organ dysfunction syndrome. |
| IV | AGI has progressed to become directly and immediately life-threatening, with worsening of multiple organ dysfunction syndrome and shock. Examples: bowel ischemia with necrosis, GI bleeding leading to hemorrhagic shock, Ogilvie syndrome, abdominal compartment syndrome requiring decompression |

**Supplemental Table S2** Summary of Empirical Treatment with Antibacterial Drugs for Bloodstream Infections

| Type of Infection / Associated Condition | Pathogens | First - line Treatment | Alternative Treatment |
| --- | --- | --- | --- |
| Primary bloodstream infection (sepsis) | MRSA, MRSCoN | Vancomycin 1g iv q12h, or Norvancomycin 0.8g iv q12h, or Teicoplanin (loading dose 6mg/kg q12h for 3 doses, maintenance dose 6mg/kg iv qd) | Daptomycin 6mg/kg iv qd |
|  | MSSA, MSSCoN | Oxacillin 2g iv q4h, or Cefazolin 2g iv q8h | Vancomycin 1g iv q12h, or Norvancomycin 0.8g iv q12h, or Teicoplanin (loading dose 6mg/kg q12h for 3 doses, maintenance dose 6mg/kg iv qd) |
|  | Enterococcus | Vancomycin 15 - 20mg/kg iv q8 - 12h; or Teicoplanin (loading dose 6mg/kg q12h for 3 doses, maintenance dose 6mg/kg iv qd) | Penicillin 3.2 million U iv q4h, or Ampicillin 3g iv q6h |
| Secondary bloodstream infection (sepsis) | Community - acquired pneumonia: Streptococcus pneumoniae, Haemophilus influenzae, Moraxella catarrhalis | Ceftriaxone 1 - 2g iv q24h + Azithromycin 500mg iv qd or respiratory quinolones (Levofloxacin 750mg iv qd, or Moxifloxacin 400mg iv qd) | Ertapenem 1g iv q24h + Azithromycin 500mg iv qd |
|  | Hospital - acquired pneumonia: MRSA | Anti - MRSA drugs (Vancomycin, or Linezolid, or Teicoplanin, or Norvancomycin) | Same as the anti - MRSA drugs on the left |
|  | Catheter - related: Staphylococcus epidermidis, Staphylococcus aureus (immunocompetent) | Oxacillin or Cefazolin for methicillin - sensitive strains | Vancomycin 1g iv q12h, or Norvancomycin, or Teicoplanin for methicillin - resistant strains |
|  | Catheter - related: Staphylococcus epidermidis, MRSA/MSSA (immunocompromised) | Vancomycin 1g iv q12h or aminoglycosides | Vancomycin + anti - pseudomonal carbapenems |
|  | Peritonitis: Enterobacteriaceae, Bacteroides, Fusobacterium, Enterococcus | Piperacillin/Tazobactam 4.5g iv q8 - 6h, Cefoperazone/Sulbactam 3.0g iv q12 - 8h, or Ertapenem 1g iv qd | Imipenem/Cilastatin 0.5g iv q6h, or Meropenem 1 - 2g iv q8h, or Panipenem/Betamipron 1.0g iv q12h, or 0.5g iv q6h, or Biapenem 0.6g iv q12 - 8h |
|  | Cholecystitis, cholangitis: Enterobacteriaceae, Enterococcus, Bacteroides, Bacillus, rarely Candida | Cefoperazone/Sulbactam 3.0g iv q12 - 8h, or Piperacillin/Tazobactam 4.5g iv q8 - 6h, or Ertapenem 1g iv qd | Imipenem/Cilastatin 0.5g iv q6h, or Meropenem 1 - 2g iv q8h, or Panipenem/Betamipron 1.0g iv q12h, or 0.5g iv q6h, or Biapenem 0.6g iv q12 - 8h |
|  | Urinary tract infection: Enterobacteriaceae (Escherichia coli), Pseudomonas aeruginosa, Enterococcus, rarely Staphylococcus aureus | Piperacillin/Tazobactam 4.5g iv q8 - 6h, or carbapenems such as Imipenem/Cilastatin 0.5g iv q6h, Meropenem 1 - 2g iv q8h, Panipenem/Betamipron 1.0g iv q12h, or 0.5g iv q6h, Biapenem 0.6g iv q12 - 8h | Fluoroquinolones such as Ciprofloxacin 400mg iv q12h, or Levofloxacin 750mg iv qd |
| Other related infections | Indwelling intravenous catheter infection: MRSA/MSSA, Staphylococcus epidermidis | Vancomycin 1g iv q12h or Norvancomycin 0.8g iv q12h |  |
|  | Tunnel - type intravenous catheter and infusion device infection, double - lumen hemodialysis catheter infection: Staphylococcus epidermidis, Staphylococcus aureus, rarely: Leuconostoc or Lactobacillus | Vancomycin 1g iv q12h or Norvancomycin 0.8g iv q12h |  |
|  | Fat emulsion infusion - related infection: Staphylococcus epidermidis | Vancomycin 1g iv q12h or Norvancomycin 0.8g iv q12h |  |
|  | Prevention of infection in long - term use of intravenous catheters | 1. Try to choose the subclavian vein for intubation, avoid the femoral vein; 2. Must wash hands standardizedly and effectively; 3. Strict aseptic operation; 4. Use 2% chlorhexidine to disinfect the skin as much as possible | Catheters soaked in chlorhexidine or sulfadiazine, or minocycline or rifampicin |
|  | Pacemaker or implantable cardioverter - defibrillator infection (infection at the implantation site, endocarditis): Staphylococcus aureus, Staphylococcus epidermidis, Gram - negative bacilli, fungi | Device removal + Vancomycin 1g iv q12h or Norvancomycin 0.8g iv q12h + Rifampicin | Device removal + Daptomycin + Rifampicin |
|  | Ventricular assist device (wire, artificial heart - related infection): Staphylococcus aureus, Staphylococcus epidermidis, | Vancomycin 1g iv q12h or Norvancomycin 0.8g iv q12h + Ciprofloxacin, or Levofloxacin | Daptomycin can be used instead of vancomycin or norvancomycin, and Cefepime can be used instead of fluoroquinolones, |
|  | Bacterial infectious cavernous sinus thrombosis: Staphylococcus aureus, Group A Streptococcus, Haemophilus influenzae | Vancomycin 1g iv q12h or Norvancomycin 0.8g iv q12h + Ceftriaxone | Linezolid 600mg iv q12h + Ceftriaxone |
|  | Infective endocarditis (native valve, non - drug user): Streptococcus viridans, other Streptococci, Staphylococcus, Enterococcus; rarely fungi | Penicillin 3.2 million U iv q4h, or Ampicillin 2g iv q4h + Oxacillin 2g iv q4h for 6 - 8 weeks + Gentamicin 1mg/kg iv/im q8h for 7 - 14d |  |
|  | Infective endocarditis (native valve, drug user): mainly Staphylococcus aureus, rarely other bacteria | Vancomycin 1g iv q12h, for those with weight > 100kg, 1.5g iv q12h; or Norvancomycin 0.8g iv q12h for 6 - 8 weeks | Daptomycin |
|  | Infective endocarditis (early prosthetic valve, post - operation < 2 months): Staphylococcus epidermidis, Staphylococcus aureus; rarely Enterobacteriaceae, fungi | Vancomycin 1g iv q12h + or Norvancomycin 0.8g iv q12h + Gentamicin 1mg/kg iv/im q8h + Rifampicin 600mg po qd (pay attention to monitoring renal function) | Daptomycin |
|  | Infective endocarditis (early prosthetic valve, post - operation > 2 months): Staphylococcus epidermidis, Streptococcus viridans; Enterococcus, Staphylococcus aureus |  |  |

**Supplemental Table S3** Prevention and Control Strategies for Bloodstream Infections

| Stages of Prevention and Control | Specific Measures |
| --- | --- |
| Preadministration Assessment | 1. Strictly master the indications for catheterization and avoid unnecessary catheter indwelling. 2. Evaluate the patient's infection risk factors (such as immune status, skin condition). 3. Select catheters with the least number of lumens and the smallest diameter, and preferentially use CVC or PICC with antibacterial coating. 4. Conduct special training for medical staff (aseptic operation, infection prevention and control knowledge). |
| Control during Catheterization | 1. Maximum sterile barrier: Operators wear sterile surgical gowns, gloves, masks, and hats; patients are fully covered with sterile drapes; ultrasound probes are covered with sterile protective sleeves. 2. Skin disinfection: Disinfect with 2% chlorhexidine gluconate alcohol solution, with a range of ≥8cm, and let it dry naturally. 3. Selection of puncture site: For adults, the subclavian vein is preferred, followed by the internal jugular vein, and the femoral vein should be avoided; for PICC, the basilic vein is preferred. 4. Ultrasound guidance: Improve the success rate of puncture and reduce the risk of mechanical damage and infection. |
| Post-catheterization Maintenance | 1. Dressing management: Sterile transparent dressings are changed every 7 days, and gauze dressings are changed every 2 days; dressings are changed immediately when loose or contaminated; chlorhexidine antibacterial dressings can be used for patients over 18 years old. 2. Flushing and sealing the catheter: Pulse flushing + positive pressure sealing, using single-dose flushing and sealing solution; fully flush the catheter after blood transfusion or administration of special drugs. 3. Replacement of infusion devices: Infusion sets are replaced every 24 hours for continuous infusion, and blood transfusion sets are replaced every 4 hours; infusion connectors are replaced immediately when contaminated or with residues. 4. Daily assessment: Evaluate the necessity of the catheter and remove unnecessary catheters as early as possible. |
| Bundle Intervention Strategies | 1. Hand hygiene: Strictly implement the seven-step handwashing method or use instant hand disinfectants before and after contacting the catheter. 2. Daily assessment of indications for catheter removal: Avoid unnecessary catheter indwelling. 3. Standardized operation checklist: Ensure that the catheterization and maintenance links comply with specifications. 4. Multidisciplinary collaboration: Establish an infection prevention and control team, regularly monitor the infection rate and feedback improvement measures. |
| Post-infection Control and Prevention Measures | 1.Peripheral venous catheters (PVCs) must be removed immediately with infusion discontinued, while PICCs, CVCs, or ports may be temporarily retained pending physician-ordered blood cultures. 2.Catheter removal is mandatory for Staphylococcus aureus infections or complications like tunnel infection/septic thrombosis. 3.Targeted antimicrobial therapy should be initiated based on microbiological results. 4.Post-removal protocols require catheter integrity inspection, with PICC/CVC sites maintaining occlusive dressings for 24 hours. 4.Patients should be closely monitored for fever and signs of local infection (erythema, swelling, pain) at insertion sites, with prompt documentation and intervention for any abnormalities. |
| Management of Special Scenarios | 1. Emergency catheterization: If aseptic operation cannot be guaranteed, remove the catheter within 48 hours and re-catheterize at a different site. 2. Blood purification catheters: The right internal jugular vein is preferred, and the femoral vein should be avoided; use a closed continuous flushing system. |
| Monitoring and Feedback | 1. Targeted monitoring: Regularly count the incidence of CRBSI, analyze the source of infection and high-risk links. 2. Continuous quality improvement: Optimize the prevention and control process through PDCA cycle, such as improving hand hygiene compliance and standardizing the frequency of dressing changes. 3. Patient education: Inform patients of the key points of catheter maintenance (such as avoiding contact with water, reporting abnormalities in time). |

**Supplemental Table S4** Antibiotic Resistance in Pathogens to Different Antibiotics (%)

| Antibiotics | Total(n=126) | Hospital-acquired(n=100) | Community-acquired(n=26) |
| --- | --- | --- | --- |
| Penicillin antibiotics | 116 (92.1) | 98 (98.0) | 18 (69.2) |
| Tetracycline antibiotics | 51 (40.5) | 39 (39.0) | 12 (46.2) |
| Aminoglycoside antibiotics | 69 (54.8) | 62 (62.0) | 7 (26.9) |
| Quinolone antibiotics | 100 (79.4) | 88 (88.0) | 12 (46.2) |
| Macrolide antibiotics | 97(77.0) | 82 (82.0) | 15 (57.7) |
| Linezolid | 5(3.2) | 4(4.0) | 1(3.8) |
| Tigecycline | 9(7.1) | 9(9.0) | 0 |
| Vancomycin | 7(5.6) | 6(6.0) | 1(3.8) |

**Supplemental Table S5.** Analysis of the drug resistance patterns in strains isolated from community-acquired

| Antibiotics | MRSA(n=1) | MSSA(n=3) | MRCNS(n=9) | MSCNS(n=5) | Enterococcus faecium(n=5) | Enterococcus faecalis(n=2) |
| --- | --- | --- | --- | --- | --- | --- |
| Penicillin G | 1(100) | 3(100) | 9 (100) | 4(80.0) | 5(100) | 0 |
| Oxacillin | 1(100) | 0 | 9 (100) | 4(80.0) | 5(100) | 1(50.0) |
| Ampicillin | 0 | 0 | 7 (77.8) | 3(60.0) | 5(100) | 0 |
| Chloramphenicol | 1(100) | 0 | 8 (88.9) | 3(60.0) | 5(100) | 1(50.0) |
| Ciprofloxacin | 0 | 1(33.3) | 5 (55.6) | 4(80.0) | 5(100) | 1(50.0) |
| Levofloxacin | 0 | 1(33.3) | 4 (44.4) | 4(80.0) | 5(100) | 0 |
| Moxifloxacin | 0 | 3(100) | 4 (44.4) | 2(40.0) | 1(20.0) | 0 |
| Rifampicin | 1(100) | 0 | 0 | 0 | 4(80.0) | 1(50.0) |
| SMZ | 0 | 0 | 4 (44.4) | 1 (20.0) | 4(80.0) | 1(50.0) |
| Clindamycin | 0 | 0 | 6 (66.7) | 2(40.0) | 5(100) | 1(50.0) |
| Erythrocin | 1(100) | 0 | 8 (88.9) | 3(60.0) | 5(100) | 2(100) |
| Streptomycin | 0 | 0 | 7 (77.8) | 3(60.0) | 3(60.0) | 2(100) |
| Vancomycin | 1(100) | 0 | 0 | 0 | 0 | 0 |
| Quinupristin/  Dalfopristin | 1(100) | 0 | 0 | 0 | 0 | 2(100) |
| Gentamicin | 0 | 0 | 3 (33.3) | 3(60.0) | 4(80.0) | 1(50.0) |
| Linezolid | 0 | 0 | 1 (11.1) | 0 | 0 | 0 |
| Tetracycline | 0 | 2(66.7) | 4 (44.4) | 0 | 3(60.0) | 2(100) |
| Tigecycline | 0 | 0 | 0 | 0 | 0 | 0 |

infections

**Supplemental Table S6.** Analysis of the drug resistance patterns in strains isolated from hospital-acquired infections

| Antibiotics | MRSA(n=4) | MSSA(n=1) | MRCNS(n=44) | MSCNS(n=18) | Enterococcus faecium(n=28) | Enterococcus faecalis(n=4) |
| --- | --- | --- | --- | --- | --- | --- |
| Penicillin G | 4(100) | 1(100) | 43 (97.7) | 17 (94.4) | 26 (92.9) | 1 (25.0) |
| Oxacillin | 4(100) | 0 | 41 (93.2) | 12 (66.7) | 25 (89.3) | 2 (50.0) |
| Ampicillin | 3(75.0) | 0 | 31 (70.5) | 8 (44.4) | 25 (89.3) | 1 (25.0) |
| Chloramphenicol | 2(50.0) | 0 | 29 (65.9) | 9 (50) | 24 (85.7) | 3 (75.0) |
| Ciprofloxacin | 4(100) | 0 | 38 (86.4) | 12 (66.7) | 27 (96.4) | 2 (50.0) |
| Levofloxacin | 4(100) | 0 | 39 (88.6) | 10 (55.6) | 26 (92.9) | 2 (50.0) |
| Moxifloxacin | 4(100) | 1(100) | 31 (70.5) | 5 (27.8) | 27 (96.4) | 2 (50.0) |
| Rifampicin | 4(100) | 0 | 12 (27.3) | 4 (22.2) | 23 (82.1) | 2 (50.0) |
| SMZ | 1(25.0) | 0 | 18 (40.9) | 7 (38.9) | 23 (82.1) | 2 (50.0) |
| Clindamycin | 3(75.0) | 0 | 29 (65.9) | 8 (44.4) | 24 (85.7) | 2 (50.0) |
| Erythrocin | 3(75.0) | 0 | 34 (77.3) | 10 (55.6) | 27 (96.4) | 2 (50.0) |
| Streptomycin | 3(75.0) | 0 | 29 (65.9) | 7 (38.9) | 16 (57.1) | 2 (50.0) |
| Vancomycin | 4(100) | 0 | 1 (2.3) | 0 | 1 (3.6) | 0 |
| Quinupristin/  Dalfopristin | 4(100) | 0 | 6 (13.6) | 1 (5.6) | 2 (7.1) | 4(100) |
| Gentamicin | 4(100) | 0 | 25 (56.8) | 9 (50) | 17 (60.7) | 2 (50.0) |
| Linezolid | 0 | 0 | 3 (6.8) | 0 | 1 (3.6) | 0 |
| Tetracycline | 3(75.0) | 0 | 15 (34.1) | 2 (11.1) | 16 (57.1) | 3 (75.0) |
| Tigecycline | 1(25.0) | 0 | 5 (11.4) | 1 (5.6) | 2 (7.1) | 0 |

**Supplemental Table S7** Association between AGI and mortality risk of overall GPB-BSI using Fine & Gray models for competing risk.

| Groups | N(%) | Crude | Adjust |
| --- | --- | --- | --- |
|  |  | HR(95%CI) | HR(95%CI) |
| AGI | 10(71.4) | 3.29  (1.65~6.56)* | 3.94  (1.96~7.92)* |
| Subgroups |  |  |  |
| AGI 0 | 32(31.1) | 1(Ref) | 1(Ref) |
| AGI I-II | 4(57.1) | 2.59 (1.02~6.58)*** | 2.85  (1.20~6.77)*** |
| AGI III-IV | 6(85.7) | 6.30  (3.42~11.62)* | 6.36  (2.68~15.09)* |
| P-for trend |  | <0.001 | <0.001 |

Notes: **p*≤0.001,***p*<0.010,****p*<0.050；Adjust for age, gender, comorbidities(digestive diseases, cancer, type 2 diabetes),MDROs, antibiotic combination(>3 types), irrational antibiotic therapy, mechanical ventilation (<72 hours), infection timing, long-term bedridden patients, long term hospitalization, acute respiratory failure , acute heart failure, acute renal failure, septic shock.

**Supplemental Table S8** Multivariable COX analysis between AGI grades and 30-day-death in subgroup among older patients with GPB-BSI

| Subgroup | Variable | Events(%) | HR(95%CI) | P for interaction |
| --- | --- | --- | --- | --- |
| Age |  |  |  | 0.662 |
| <75 | AGI I-II | 2 (100) | 3.41 (0.37~31.46) |  |
|  | AGI III-IV | 1 (100) | 12.94 (0.9~185.4) |  |
| >=75 | AGI I-II | 4 (80.0) | 3.17 (0.7~14.3) |  |
|  | AGI III-IV | 5 (83.3) | 12.86 (2.56~64.63) |  |
| Gender |  |  |  | 0.073 |
| Female | AGI I-II | 1 (50.0) | 3.82 (0.27~54.52) |  |
|  | AGI III-IV | 0 (0) | 0 (0~Inf) |  |
| Male | AGI I-II | 5 (100) | 3.35 (1.13~9.93) |  |
|  | AGI III-IV | 6 (100) | 8.2 (2.43~27.64) |  |
| Digestive diseases |  |  |  | 0.589 |
| No | AGI I-II | 4 (100) | 3.92 (0.97~15.9) |  |
|  | AGI III-IV | 4 (100) | 13.79 (2.51~75.67) |  |
| Yes | AGI I-II | 2 (66.7) | 1.81 (0.25~12.85) |  |
|  | AGI III-IV | 2 (66.7) | 4.65 (0.67~32.38) |  |
| MDRS |  |  |  | 0.535 |
| No | AGI I-II | 3 (75.0) | 2.6 (0.5~13.46) |  |
|  | AGI III-IV | 5 (100) | 2.6 (0.54~12.54) |  |
| Yes | AGI I-II | 3 (100) | 3.84 (0.83~17.8) |  |
|  | AGI III-IV | 1 (50.0) | 2.8 (0.26~30.62) |  |
| SOFA |  |  |  | 0.493 |
| <11 | AGI I-II | 1 (100) | 4.16 (0.32~54.71) |  |
|  | AGI III-IV | 3 (75.0) | 12.08 (2.41~60.54) |  |
| >=11 | AGI I-II | 5 (83.3) | 3.67 (0.81~16.57) |  |
|  | AGI III-IV | 3 (100) | 3.16 (0.39~25.49) |  |
